# Emerging of H5N6 Subtype Influenza Virus with 129-Glycosylation Site on Hemagglutinin in Poultry in China Acquires Immune Pressure Adaption

**DOI:** 10.1128/spectrum.02537-21

**Published:** 2022-04-21

**Authors:** Nuo Xu, Yuwei Wu, Yulian Chen, Yue Li, Yuncong Yin, Sujuan Chen, Huiguang Wu, Tao Qin, Daxin Peng, Xiufan Liu

**Affiliations:** a College of Veterinary Medicine, Yangzhou University, Yangzhou, Jiangsu, China; b Jiangsu Co-Innovation Center for the Prevention and Control of Important Animal Infectious Disease and Zoonoses, Yangzhou, Jiangsu, China; c The International Joint Laboratory for Cooperation in Agriculture and Agricultural Product Safety, Ministry of Education, Yangzhou University, Yangzhou, Jiangsu, China; d Jiangsu Research Centre of Engineering and Technology for Prevention and Control of Poultry Disease, Yangzhou, Jiangsu, China; Wuhan Institute of Virology

**Keywords:** H5N6, antigenic drift, avian influenza virus, glycosylation, vaccination

## Abstract

For an investigation into the effects of glycosylation site modification on hemagglutinin (HA) on the biological characteristics of the H5N6 subtype avian influenza virus (AIV), the HA sequences of H5N6 AIVs from Global Initiative on Sharing All Influenza Data (GISAID) and the isolates in China were analyzed for genetic evolution and glycosylation site patterns. Eight recombinant H5N6 AIVs with different glycosylation site patterns were constructed, and their biological characteristics were determined. The results showed that H5N6 AIVs containing a 129-glycosylation site on HA are becoming prevalent strains in China. Acquisition of the 129-glycosylation site on the HA of H5N6 AIVs increased thermostability, decreased pH stability, and attenuated pathogenicity and contact transmission in chickens. Most importantly, H5N6 AIVs escaped the neutralization activity of the Re-8-like serum antibody. Our findings reveal that H5N6 AIVs containing the 129-glycosylation site affect antigenicity and have become prevalent strains in China.

**IMPORTANCE** H5N6 avian influenza viruses (AIVs) were first reported in 2013 and have spread throughout many countries. In China, compulsory vaccine inoculation has been adopted to control H5 subtype avian influenza. However, the effect of vaccination on the antigenic drift of H5N6 AIVs remains unknown. Here, we found that H5N6 AIVs with the 129-glycosylation site on hemagglutinin were the dominant strains in poultry in China. The neutralization assay of the serum antibody against the H5 subtype vaccine Re-8 showed a significantly lower neutralization activity against H5N6 AIVs with the 129-glycosylation site compared to that against H5N6 AIVs without the 129-glycosylation site, indicating that the 129-glycosylation site may be a crucial molecular marker for immune evasion.

## INTRODUCTION

The H5N6 avian influenza virus (AIV) broke out in China in 2013 and then spread throughout Asia (1). H5N1 AIVs have been gradually replaced by the prevalence of H5N6 AIVs in poultry (2). Compared with the pathogenicity of H5N1 AIVs, the H5N6 virus maintained high pathogenicity in chickens and caused the infection and death of waterfowl (3, 4). Moreover, outbreaks of H5N6 AIVs in wild birds have been reported in South Korea, Japan, and China, placing severe pressure on the prevention and control of H5N6 AIVs (4, 5).

Hemagglutinin (HA) is the main antigenic protein on the surface of AIVs and is responsible for binding sialic acid receptors (6). AIVs may acquire adaptions by modifying HA protein characteristics, including pH stability, thermostability, receptor binding activity, and antigenic drift (7, 8). The addition of glycosylation sites on HA is an important mechanism that contributes to antigenic drift (9). In general, the glycosylation sites in the stem region of the HA protein are conserved, whereas the glycosylation sites on the head of the HA protein vary in position and number (10, 11). The head glycosylation site of the HA protein not only affects receptor binding properties and regulates virulence (12, 13) but also assists the virus in escaping antibody pressure (14). In China, compulsory vaccine inoculation has been adopted to control H5 AIVs since 2004, and the vaccine has been continually updated. Re-5 vaccine based on clade 2.3.4 was used from 2008 to 2014, Re-8 vaccine based on the clade 2.3.4.4e was used from 2014 to 2018, and Re-11 vaccine based on the clade 2.3.4.4d was used from 2019 to now (15). The virus isolation rate of H5 AIVs decreased after the implementation of the vaccine update and “1110” strategy for live poultry markets (16), indicating that the vaccine plays a significant role in the prevention and control of H5 AIVs. However, vaccination pressure inevitably accelerates the evolution of the influenza virus (17–20). The 158-glycosylation site on the HA of H5 AIVs can affect antigenicity (21, 22), and the 131-glycosylation site on the HA of the clade 7.2 H5N1 virus contributes to antigen drift (23). Because of the prevalence of H5N6 AIVs in China, there are some emerging glycosylation sites on HA; thus, further study is necessary on the effect of these new glycosylation sites on their biological characteristics.

In this study, we analyzed the glycosylation site pattern on HA of H5N6 AIVs and constructed a series of recombinant viruses with glycosylated or deglycosylated sites at residues 63 and/or 129 on the HA protein and evaluated their antigen variation, thermostability, pH stability, pathogenicity, and contact transmission in chickens.

## RESULTS

### H5N6 AIV with a 129-glycosylation site on HA became a prevailing strain in China.

**FIG 1:**
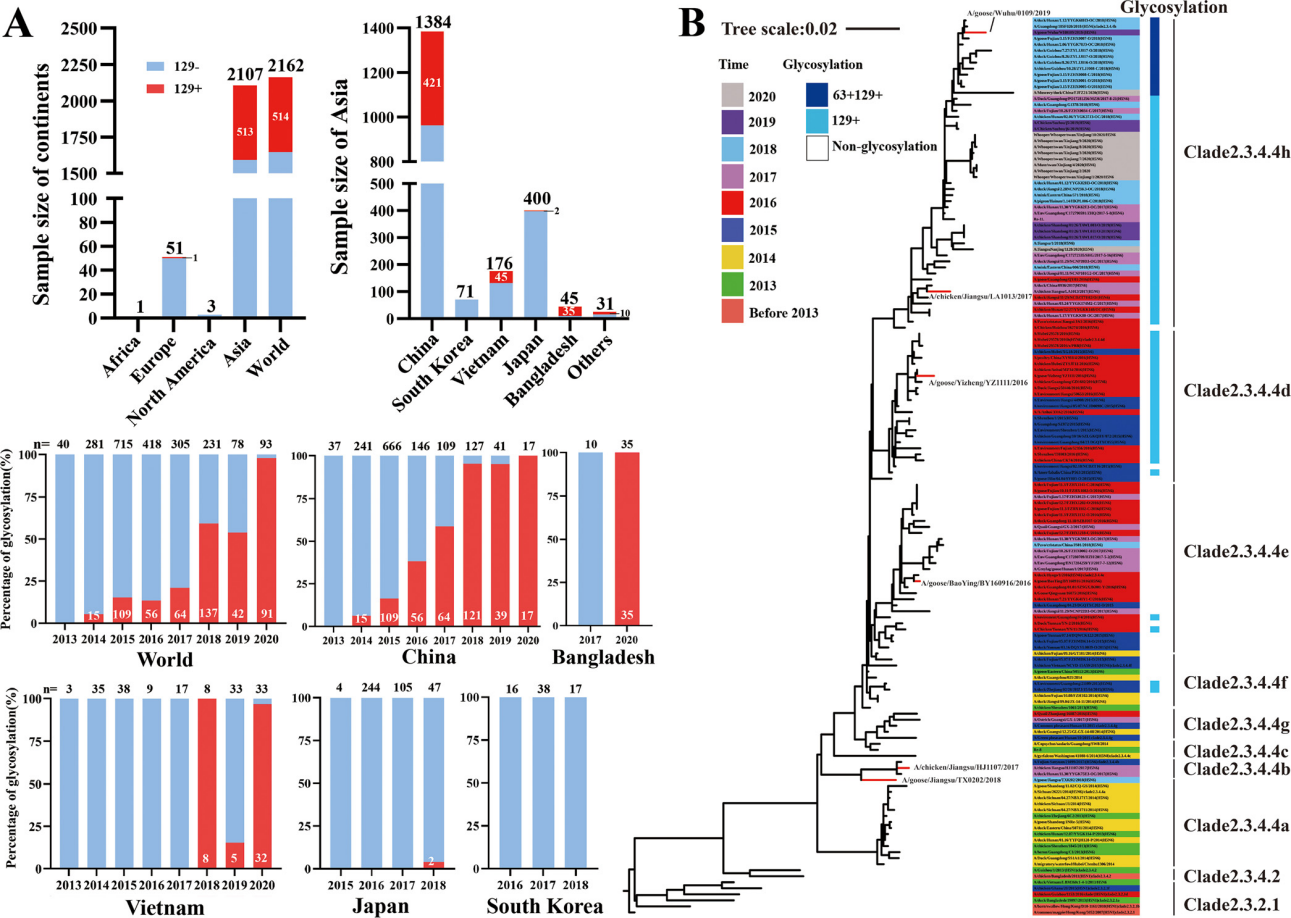
Isolate rates (A) and HA evolution analysis (B) of H5N6 AIVs. Isolation rates of H5N6 AIVs with the 129-glycosylation site on HA protein worldwide and in Asia are shown. Sample size per year is listed at the top of the picture in black text; strains with 129-glycosylation site are marked with white at the bottom of the columns or in black on the right side of column (A). HA sequences of H5N6 AIVs in China were downloaded from GISAID (*n* = 160); the phylogenetic tree of HA genes is drawn according to isolation years and classified as different subclades; the 129- and 63-glycosylation sites are marked in light and dark blue, respectively; the viruses used in this study are marked in red in the phylogenetic tree (B).

A total of 2,162 HA sequences of H5N6 AIVs were collected from Global Initiative on Sharing All Influenza Data (GISAID), in which most isolates were distributed in Asia (*n* = 2,107), followed by Europe (*n* = 51), and there were a few isolates in Africa (*n* = 1) and North America (*n* = 3). Analysis of the HA sequences of H5N6 viruses from Asian countries revealed that the number of H5N6 AIVs with a 129-glycosylation site on HA isolated in China was the highest (*n* = 421); however, a small number of H5N6 viruses with the 129-glycosylation site were isolated from Bangladesh (*n* = 35), Vietnam (*n* = 45), Japan (*n* = 2), and other countries (*n* = 10) (Fig. 1A). Further analysis indicated that H5N6 AIVs with a 129-glycosylation site appeared in the world in 2014, and the proportion reached nearly 100% in 2020. In China, H5N6 AIVs with the 129-glycosylation site were first isolated in 2014, and the proportion of these viruses has increased annually, reaching 100% by 2020. However, H5N6 AIVs with the 129-glycosylation site accounted for 0.5% in Japan, and no occurrence was observed in South Korea. Since 2018, AIVs with the 129-glycosylation site were isolated in Vietnam and were 25.6% of all isolates. H5N6 AIVs with the 129-glycosylation site were isolated in Bangladesh in 2020, and the proportion of all isolates was 77.8% (Fig. 1A). H5N6 viruses with a 63-glycosylation site on HA were isolated in 2018, and the proportion of H5N6 viruses with the 63-glycosylation site was 5.4% among global H5N6 viruses and 5.0% among H5N6 viruses from China (Fig. S1). H5N6 viruses with the 129-glycosylation site were distributed in clade 2.3.4.4d and clade 2.3.4.4h, whereas H5N6 viruses with both 63- and 129-glycosylation sites appeared in clade 2.3.4.4h (Fig. 1B). The Re-8 vaccine strain showed no glycosylation site at residues 63 or 129 on HA; however, the Re-11 vaccine strain showed a 129- but not a 63-glycosylation site on HA (Table 1). Hence, H5N6 AIVs with the 129-glycosylation site have become prevalent strains in China according to the GISAID database.

**TABLE 1:** Glycosylation site patterns at residues 63 and 129 on HA of H5N6 AIVs

Virus name	Virus source	Clade	Glycosylation^*a*^ status at site:	
			63	129
A/chicken/Anhui/1/2006(H5N1) (Re-5)	Vaccine strain	2.3.4	−	−
A/Sichuan/26221/2014(H5N6)	WHO reference	2.3.4.4a	−	−
A/Fujian-Sanyuan/21099/2017(H5N6)	WHO reference	2.3.4.4b	−	−
A/chicken/Jiangsu/HJC1107/2017(H5N6)	This study	2.3.4.4b	−	−
A/goose/Jiangsu/TXG0202/2018(H5N6)	This study	2.3.4.4b	−	−
A/chicken/Guizhou/4/2013(H5N1) (Re-8)	Vaccine strain	2.3.4.4e	−	−
A/duck/Hyogo 1/2016 (H5N6)	WHO reference	2.3.4.4e	−	−
A/goose/BaoYing/BY0916/2016(H5N6) (B)	This study	2.3.4.4e	−	−
A/chicken/Vietnam/NCVD 2015(H5N6)	WHO reference	2.3.4.4f	−	−
A/Hubei/29578/2016b(H5N6)	WHO reference	2.3.4.4d	−	+
A/goose/Yizheng/YZG1111 2016(H5N6)	This study	2.3.4.4d	−	+
A/duck/Guizhou/S4184/2017 (H5N6) (Re-11)	Vaccine strain	2.3.4.4d	−	+
A/chicken/Jiangsu/LAC1013/2017(H5N6)	This study	2.3.4.4h	−	+
A/Guangdong/18SF020/2018(H5N6)-like	WHO reference	2.3.4.4h	+	+
A/goose/Wuhu/WH0109/2019/(H5N6) (W)	This study	2.3.4.4h	+	+

a +, glycosylated; −, nonglycosylated.

### The 129-glycosylation site on HA protein decreased low-pH stability but enhanced thermostability of H5N6 AIVs.

**FIG 2:**
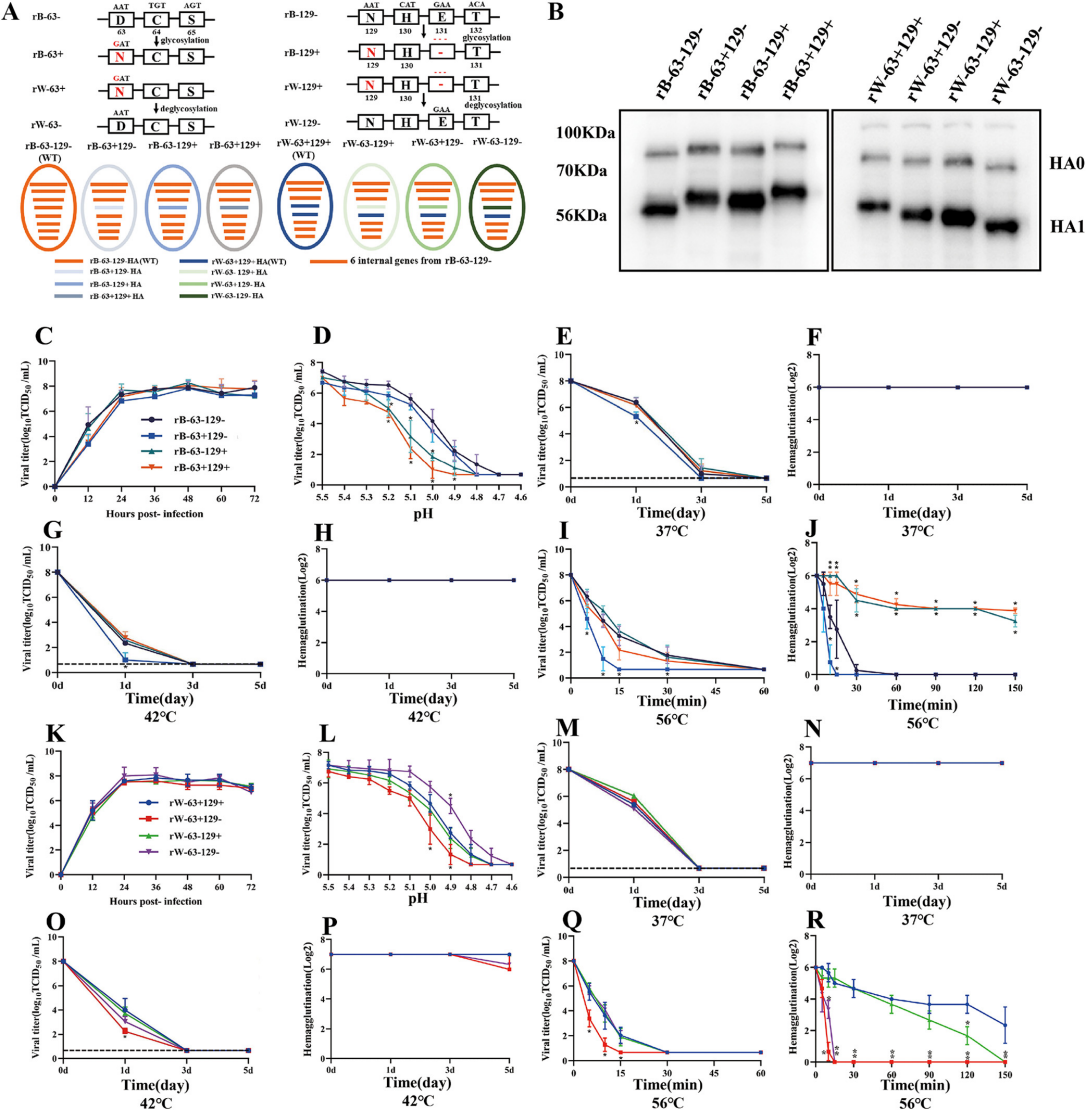
Construction of recombinant viruses with different glycosylation site patterns on HA protein (A and B) and determination of their growth curve (C and K), low-pH stability (D and L), and thermostability (E to J and M to R) for rB group (C to J) and rW group (K to R). rB-63−129−, rW-63+129+; their HA mutant viruses were generated by reverse genetics based on the HA glycosylation and deglycosylation strategy (A). Whole-cell lysates obtained from MDCK cells infected with recombinant viruses at an MOI of 1 for 12 h were subjected to Western blotting with anti-H5 HA protein mouse monoclonal antibodies (B). Growth curves of recombinant viruses in CEF were determined by TCID_50_ (C and K). Low-pH stability of the recombinant viruses at different pH values adjusted in 0.1-unit increments from 4.6 to 5.6 was determined by TCID_50_ titers (D and L). Thermostability of recombinant viruses at 37°C, 42°C, and 56°C were determined by TCID_50_ titers and HA titers (E to J and M to R). Viral titers or HA titers of recombinant viruses were significantly different from those of the wild-type strains, *, *P* < 0.05.

Mutations in residues D63N or E131 on HA were designed to generate 63- or 129-glycosylation sites (Fig. 2A). We rescued eight recombinant AIVs with different glycosylation site patterns at residues 63 and 129 based on six internal genes from wild-type strain B. The addition of HA glycosylation sites (rB-63+129−, rB-63−129+, and rB-63+129+) decreased mobility of HA in SDS-PAGE compared to the mobility of wild-type strain rB-63−129−, and elimination of glycosylation sites (rW-63−129+, rW-63+129−, and rW-63−129−) increased HA mobility in SDS-PAGE compared to that of the wild-type strain rW-63+129+, indicating that all mutant viruses were constructed successfully (Fig. 2B). Next, we determined whether there was a growth difference among viruses with and without 63- and 129-glycosylation sites. The virus growth curve revealed no significant difference for each mutant virus *in vitro* compared to the wild-type strain rB-63−129− (Fig. 2C) or rW-63+129+ (Fig. 2K).

In the low-pH stability assay, H5N6 AIVs without both 63- and 129-glycosylation sites possessed the highest low-pH stability, and the addition of 63- and 129-glycosylation sites to HA significantly decreased its ability to tolerate low-pH stability (Fig. 2D and L). When incubated at 42°C and 56°C, the viruses with a single 63-glycosylation site showed a significant decrease in the median tissue culture infectious dose (TCID_50_; rB-63+129− and rW-63+129−), and the rB-63+129− also showed a significant decrease in the TCID_50_ at 37°C on day 1 posttreatment compared to the TCID_50_ of wild-type strains (Fig. 2E, G, I, O, and Q). Moreover, the TCID_50_ and HA titers of viruses containing the 129-glycosylation at 37°C and 42°C were similar to those of the viruses without the 129-glycosylation site (Fig. 2F, H, M, and N). However, the HA titers of viruses with the 129-glycosylation site were remarkably higher than those of viruses without the 129-glycosylation site when incubated at 56°C (Fig. 2J and R), indicating that the 129-glycosylation site was responsible for improving the thermostability of the HA proteins of H5N6 AIVs.

### The 129-glycosylation site on HA decreased both pathogenicity and contact transmission levels of H5N6 AIVs in chickens.

**FIG 3:**
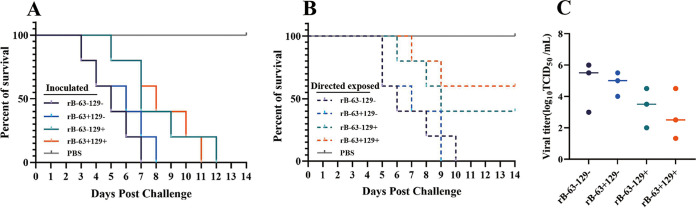
Pathogenicity of rB-63−129− and its mutants of H5N6 AIVs in chickens. Four-week-old SPF chickens were inoculated intranasally with 10^4^ EID_50_ of H5N6 AIVs in a volume of 200 μL, and the survival rates of inoculated chickens (A) and contacted chickens (B) were observed for 14 days. (C) Lung (*n* = 3) viral titers were measured on 3 dpi.

For evaluating the effect of H5N6 AIV acquiring glycosylation sites on pathogenicity and transmissibility, rB and the derived virus with 63 and/or 129 glycosylation sites were used as challenge strains. The chickens in the rB-63−129− and rB-63+129− inoculated groups died earlier than those in the rB-63−129+ and rB-63+129+ inoculated groups. Additionally, the virus containing a single 63-glycosylation site (rB-63+129−) displayed pathogenicity in chickens slightly lower than that of the wild-type rB-63−129− (Fig. 3A). The contact transmission test revealed that all chickens in rB-63+129− and rB-63−129− died within 9 days and 10 days, respectively, whereas the mortalities of the rB-63−129+ and rB-63+129+ chickens were 60% and 40%, respectively (Fig. 3B). Virus shedding in the oropharynx and cloaca of the chickens in the contact groups was detected on days 3, 5, and 7 p.i. The virus shedding ratios of chickens in the rB-63−129+ and rB-63+129+ contact groups were lower than those of chickens in the rB-63−129− and rB-63+129− contact groups (Table 2). Moreover, viruses with the 129-glycosylation site also showed decreased viral titers in the lungs of infected chickens (Fig. 3C). These results confirmed that the addition of the 129-glycosylation site on the HA of H5N6 AIVs decreased both pathogenicity and contact transmission ability in chickens.

**TABLE 2:** Viral shedding positive ratio of chickens in contacted groups

Group	Percentage of chickens with virus shedding with O or C swabs at dpc^*a*^:					
	3		5		7	
	O^*b*^	C	O	C	O	C
rB-63−129−	0% (0/5)^*c*^	0% (0/5)	100% (3/3)	33.3% (1/3)	100% (2/2)	100% (2/2)
rB-63+129−	0% (0/5)	0% (0/5)	66.7% (2/3)	33.3% (1/3)	100% (2/2)	50% (1/2)
rB-63−129+	0% (0/5)	0% (0/5)	40% (2/5)	20% (1/5)	25% (1/4)	25% (1/4)
rB-63+129+	0% (0/5)	0% (0/5)	20% (1/5)	0% (0/5)	50% (2/4)	25% (1/4)

a dpc, days postcontact.

b Oropharyngeal (O) and cloacal (C) swabs.

c Number of chickens with virus shedding/total number of chickens.

### H5N6 AIVs with the 129-glycosylation site escaped the neutralization activity of Re-8-like antisera.

**FIG 4:**
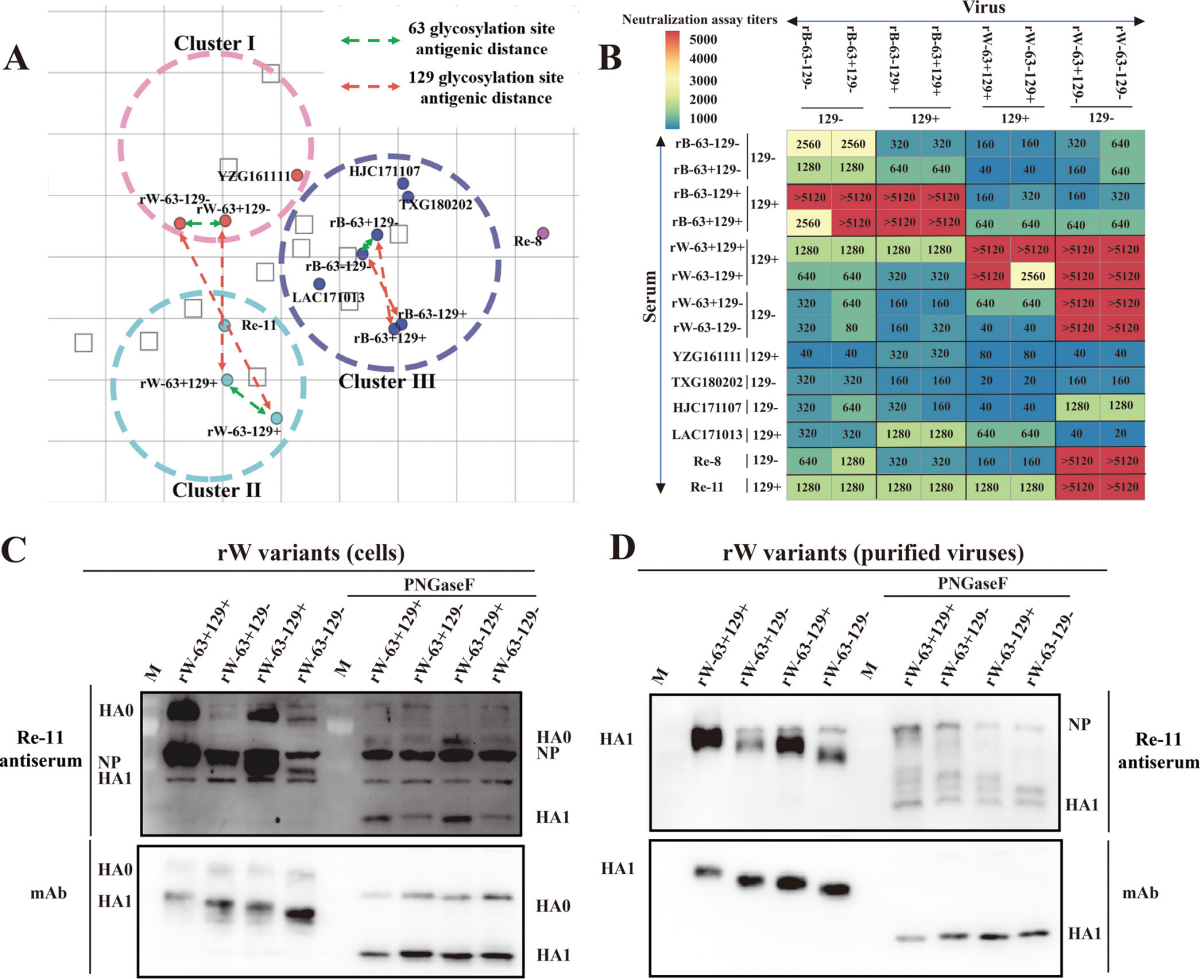
Antigenic properties of H5N6 AIVs and recombinant viruses. Antigenic cartography of H5N6 AIVs is drawn according to the HI titers of the representative H5N6 AIVs and the recombinant viruses against the antisera (A); open squares and filled circles represent antisera and viruses, respectively. Double arrows represent antigenic distances. (B) Neutralization titers of H5N6 AIVs and the recombinant viruses were detected by microneutralization assay; dark red represents high cross-neutralization reactivity, and dark blue represents low cross-neutralization reactivity. Recombinant viruses from cells lysate (C) and purified recombinant viruses from allantoic fluid (D) were analyzed by Western blotting; the same samples were treated with/without PNGase F and then Re-11 antiserum or monoclonal antibody was used as primary antibody.

For understanding the effects of glycosylation on antigenicity, eight mutated viruses and four isolated viruses with or without the 129-glycosylation site were selected (Table 1). The cross-reactivity of these viruses against each chicken antiserum was determined by a hemagglutination inhibition (HI) assay. These 12 viruses formed three antigen clusters according to their HI titers: cluster I, comprising viruses rW-63−129−, rW-63+129−, and YZG161111, cluster II, which included Re-11, rW-63+129+, and rW-63−129+, and cluster III, which included HJC171107, TXG180202, LAC171013, rB-63−129−, rB-63+129−, rB-63−129+, and rB-63+129+. Deletion of the 129-glycosylation site on the HA of rW-63+129+ and rW-63−129+ resulted in antigen drift from cluster II to cluster I. The same antigen drift was observed among rB and its mutant viruses in cluster III (Fig. 4A). Hence, the 129-glycosylation site contributes to the antigen drift of H5N6 AIVs.

Next, the neutralization titers of the antisera against the wild-type and mutant strains were determined. The neutralization titers of the antisera induced by rB-63−129− against the recombinant viruses without the 129-glycosylation site (rB-63−129− and rB-63+129−) were 2,560 and 2,560, respectively, whereas the neutralization titers against the recombinant viruses with the 129-glycosylation site (rB-63−129+ and rB-63+129+) were 320 and 320, respectively, with an 8-fold decrease. Similarly, the neutralization titers of the antisera induced by rW-63−129− against the recombinant viruses without the 129-glycosylation site (rW-63−129− and rW-63+129−) were >5,120 and >5,120, respectively, whereas the neutralization titers against the recombinant viruses with the 129-glycosylation site (rW-63−129+ and rW-63+129+) were 40 and 40, respectively, with more than a 128-fold decrease. Additionally, the antisera induced by viruses with the 129-glycosylation site (rB-63−129+, rB-63+129+, rW-63−129+, and rW-63+129+) showed good cross-neutralization titers against their derived viruses with or without the 129-glycosylation site. For antisera against the vaccine strains, the cross-neutralization titers of Re-8 antisera showed a high level against rW-63+129− and rW-63−129− (>5,120 and >5,120, respectively) and a low level against rW-63+129+ and rW-63−129+ (160 and 160, respectively), a more than 32-fold decrease. However, Re-11 antisera had similar cross-neutralization titers against rW-63+129+, rW-63−129+, rW-63−129−, and rW-63+129− (Fig. 4B). Similar results were observed in other prevailing strains with or without the 129-glycosylation site. These data confirmed that viruses with the 129-glycosylation site escape the neutralization activity of Re-8 antisera but not the neutralization activity of Re-11 antisera.

For determining whether the Re-11 antisera contained antibodies against the 129-glycosylation site on HA, the expression of HA in H5N6 AIVs was detected by Western blotting. When incubated with Re-11 antiserum, the expressions of HA proteins of the viruses with the 129-glycosylation site (rW-63+129+ and rW-63−129+) were significantly higher than those of rW-63+129− and rW-63−129− in cell lysate or purified virus (Fig. 4C and D). However, when incubated with monoclonal antibody, viruses with or without the 129-glycosylation site on HA showed similar expression of HA protein, demonstrating that the increased expression of HA protein is related to the antibody against the 129-glycosylation site on HA. Furthermore, the cell lysate or purified viruses were treated with PNGase F, an enzyme that can hydrolyze glycan, and the HA bands were significantly thinner than those of untreated viruses (Fig. 4C and D). These results confirmed that Re-11 antiserum contained an antibody targeting the 129-glycosylation site.

**FIG 5:**
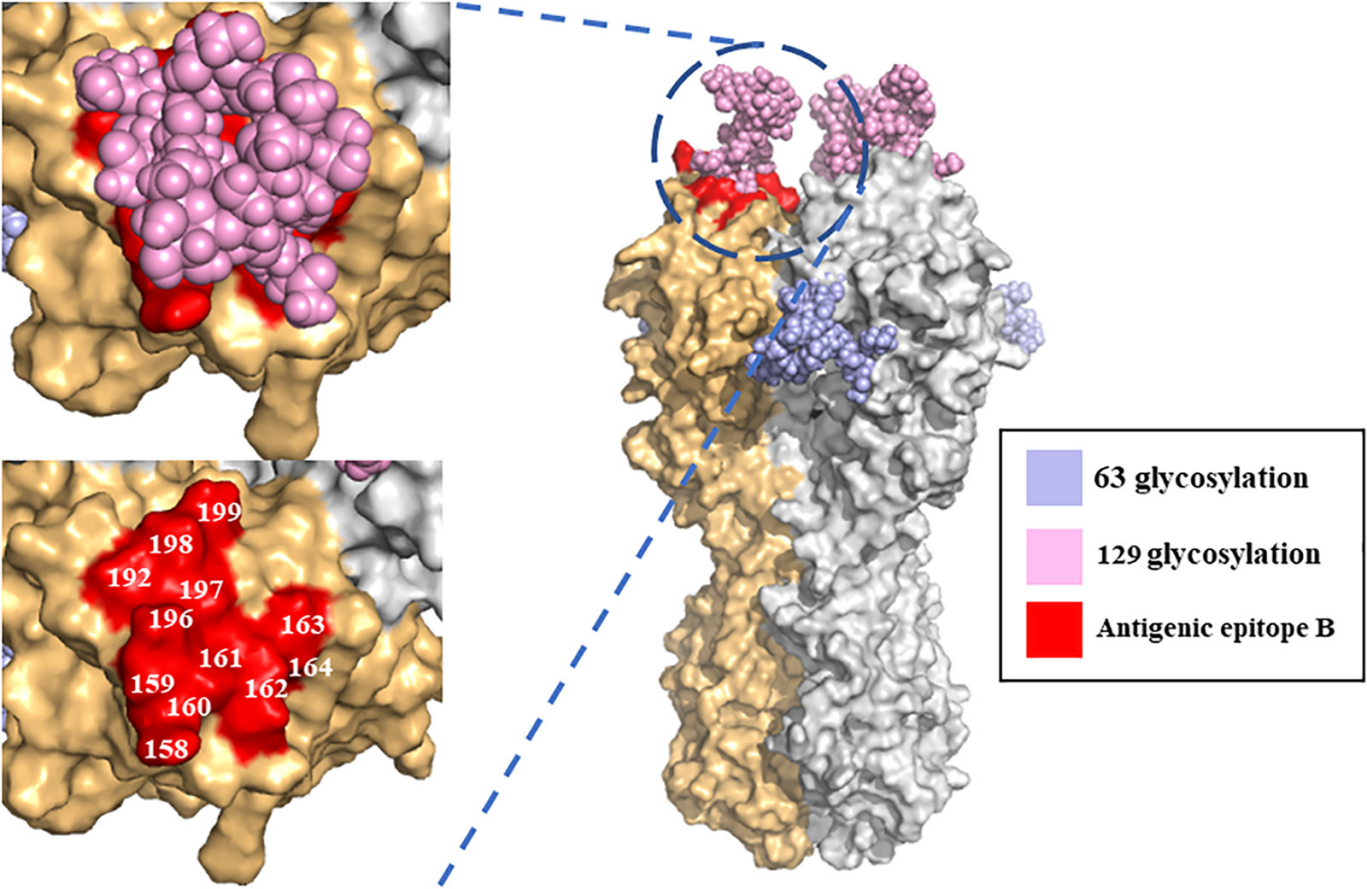
Structural modeling of N-linked glycan and antigenic epitope on HA trimers. Two monomers of the H5 HA homotrimer are shown in gray and light orange. Amino acid residues of antigenic epitope B are shown on the surface in red. The 63- and 129-glycosylation sites on HA are marked in light blue and pink, respectively.

Finally, for exploring the impact of the 129-glycosylation site on viral antigenicity from a structural perspective, a glycan model of the H5N1 virus was used, and HA trimers were generated using SWISS-MODEL (Fig. 5). The results showed that the 129-glycosylation site was projected onto the head domain of the HA protein, and the 63-glycosylation site was located in the region between the head and stem of HA. Notably, the 129-glycosylation site covers some antigenic epitope B and may induce Re-11-like antibodies against the 129-glycosylation structure and interfere with Re-8-like antibodies against antigenic epitope B.

## DISCUSSION

A study showed that more than 50% of H5N6 AIV isolates from Guangdong Province contained the D63N mutation (63-glycosylation site) and D129N/S or D130 deletion/E/T mutation (129-glycosylation) (15). Our molecular epidemiological study revealed that H5N6 viruses containing the 129-glycosylation site appeared in 2014 and became a prevailing strain in China, whereas H5N6 viruses containing the 63- and 129-glycosylation sites appeared in 2018, accounting for approximately 5.4% of all global H5N6 viruses (Fig. S1) or 28.9% of global H5N6 viruses isolated from to 2018 to 2020. Modification of the glycosylation site on the HA protein modulated the thermostability and low-pH stability of H5 AIVs. The removal of 11 glycosylation sites in the HA stem region of H5N1 AIVs decreases viral titers both at different temperatures and at a pH from 4.0 to 6.0 (24), and the deletion of 158 glycosylation sites on the HA head region enhances viral thermostability and decreases viral titers at a pH from 4.5 to 6.0 (25, 26), confirming that the changes in viral thermostability and low-pH stability caused by glycosylation modification are not always consistent. In this study, the addition of a single 63-glycosylation site on HA (rB-63+129− and rW-63+129−) decreased viral TCID_50_, both in viral thermostability at 42°C and 56°C and in low-pH stability (pH 4.5 to 5.5).

However, the addition of a 129-glycosylation site on HA improved HA thermostability at 56°C, resulting in a delayed HA titer decrease. Therefore, the emergence of the 129-glycosylation site endows the HA protein of H5N6 AIVs with the ability to maintain stability at high temperatures, which might be applied to achieve long-term preservation of the influenza vaccine. Because the temperature had no effect on the TCID_50_ of H5N6 AIVs acquiring the 129-glycosylation site compared to the TCID_50_ of their parent strain, H5N6 AIVs with the 129-glycosylation site can survive in the environment as H5N6 AIVs without the 129-glycosylation site.

An acid-stable HA protein is a molecular requirement for the pandemic influenza virus, which is necessary for pH1N1 influenza virus pathogenicity and airborne transmissibility (27). For H5 subtype AIVs, enhanced pathogenicity in chickens was found to be related to an increase in the activation of HA protein in the pH range from 5.2 to 6.0 (28, 29). This phenomenon was also observed in the H5N1 AIV infection of waterfowl; however, it was not completely consistent (30). A study also showed that an increase in the pH of the HA activation of H5N1 viruses coincides with increased sensitivity to acid inactivation (31). In this study, the sensitivity of H5N6 wild-type and mutant strains to acid inactivation was determined. The acquisition of the 129-glycosylation site on HA decreases the low-pH stability of the virus. However, the acquisition of the 129-glycosylation site on the HA of H5N6 AIVs attenuated their pathogenicity and contact transmission in chickens. Because the glycosylated recombinant virus possesses growth properties similar to those of the wild-type strains in chicken embryo fibroblast (CEF) cells, the mechanism for attenuated pathogenicity of H5N6 AIVs with the 129-glycosylation site on HA requires further investigation. Attenuation of pathogenicity by the addition of the 63- and 129-glycosylation sites on HA was also observed in the pH1N1 virus (18).

The compulsory immunization program has been implemented since 2004 in China, and the vaccination strategy has not been adopted in other Asian countries. Our molecular epidemiological study revealed that the H5N6 virus containing the 129-glycosylation site appeared and became a prevailing strain in China; however, there were few or no H5N6 viruses with the 129-glycosylation site in other Asian countries, based on the GISAID database, indicating that the H5N6 virus containing the 129-glycosylation site may be selected by vaccine pressure. Re-8-like antisera showed a high level of neutralization activity against H5N6 viruses without the 129-glycosylation site on HA and a low level of neutralization activity against H5N6 viruses with the 129-glycosylation site on HA, indicating that H5N6 viruses with the 129-glycosylation site are novel variants of the immune escape strain. Although Re-11 antisera had similar neutralization titers against rB strains with and without the 129-glycosylation site, the neutralization titers of Re-11 antisera against rW strains with the 129-glycosylation site were higher than those against rW strains without the 129-glycosylation site. Western blotting confirmed the presence of massive antibodies against the 129-glycosylation site epitope in the Re-11 antisera. The simulated 3D structure showed that the 129-glycosylation site appeared on the head domain of the HA protein and covered some area of antigenic epitope B, which may induce antibodies against the 129-glycosylation site epitope and prevent the neutralization activity of Re-8 antisera. The data suggest that the H5N6 virus with the 129-glycosylation site may be a strain that adapted under the vaccination pressure induced by the Re-8-like vaccine. Re-11 antisera had good neutralization activity against epidemic strains with the 129-glycosylation site, which probably generates novel immune escape strains under vaccination pressure.

In summary, based on GISAID data analysis, H5N6 AIVs with the 129-glycosylation site on HA are the prevailing strains in China. The additional 129-glycosylation site on the HA of H5N6 AIVs increased HA thermostability, decreased pH stability, and attenuated both pathogenicity and contact transmission levels in chickens. Thus, continuously monitoring the immune escape strain under the vaccination pressure is essential.

## MATERIALS AND METHODS

### Biosafety statement and ethical approval.

All experiments involving H5N6 AIVs were performed in animal biosafety level 3 (ABSL-3) facilities and were approved by the Biosafety Committee of Yangzhou University. All animal studies followed protocols approved by the Jiangsu Province Administrative Committee for Laboratory Animals (SYXKSU-2016-0020).

### Antibodies, virus isolation, virus purification, and virus rescue.

Commercial antisera Re-8 and Re-11 were purchased from YeBio (Qingdao, China), and polyclonal antisera against mutated viruses were collected from immunized specific-pathogen-free (SPF) chickens with inactivated viruses. Monoclonal antibodies against H5 HA protein were prepared as described previously (24).

The six H5N6 AIVs used in this study were isolated from clinical samples of diseased poultry collected between 2016 and 2020 (Table 1). The viruses were cloned by limited dilution and purified by sucrose density gradient centrifugation (32).

Two H5N6 AIVs B strains with glycosylation sites 63−129− on the HA protein and W strain with glycosylation sites 63+129+ on the HA protein were chosen for recombinant virus construction. The amplified HA genes were inserted into the pHW2000 vector and confirmed using sequence analysis. Glycosylated and deglycosylated HA plasmids were constructed using the Mut Express II fast mutagenesis kit V2 (Vazyme, China). The primers for mutation construction are listed in Table S1.

Recombinant viruses were generated according to reported protocols (33). Briefly, cocultured 293T-Madin-Darby canine kidney (MDCK) cells were transfected with rescued plasmids (six internal genes from the B strain and NA genes from the B strain or W strain) with glycosylated or deglycosylated HA plasmid. The parent strains and additional glycosylated or deglycosylated recombinant viruses rB-63−129−, rB-63+129−, rB-63−129+, rB-63+129+, rW-63+129+, rW-63−129+, rW-63+129−, and rW-63−129− were rescued.

### Phylogenetic analysis.

The HA sequences of H5N6 AIVs from different regions and countries by the end of 2020 were downloaded from GISAID (https://www.gisaid.org). Sequences with a similarity greater than 99.5% were removed using BioAider (v.1.334) (34). The selected sequence information is provided in Table S2. Phylogenetic analysis of HA genes of H5N6 AIVs was performed using IQ-TREE (v.1.6.8) (35). Different years and glycosylation sites were denoted with different markers by using ITOL (v.6.5.2) (https://itol.embl.de/).

### Western blotting and PNGase F treatment of viruses.

CEF cells were infected with recombinant viruses at a multiplicity of infection (MOI) of 1. At 24 h postinfection, the cells were lysed with 200 μL RIPA lysis buffer (Beyotime, China) on ice for 15 min, and the purified viruses were lysed using the same method. Cell lysates and purified viruses were divided into two aliquots: one was treated with 2 μL PNGase F at 37°C for 2 h, and the other was the control and treated with phosphate-buffered saline (PBS). Proteins were loaded into lanes on SDS-PAGE gels and transferred to nitrocellulose membranes. The membrane was blocked in 5% skimmed milk and incubated with Re-11 polyclonal antibody or monoclonal antibody against HA, followed by incubation with conjugated goat anti-mouse or chicken IgG antibodies (GE Whatman, England). Protein bands were detected by electrochemiluminescence (ECL; Biobest, China) (12).

### Virus growth curve, thermostability, and low-pH stability.

For detecting viral growth in CEF cells, monolayer cells were infected with each virus at an MOI of 0.001 in M199 for 1 h. The infected cells were washed with PBS, and serum‐free M199 was added. The cells were then incubated at 37°C and 5% CO_2_. The virus titers in the supernatant were monitored periodically by determining the TCID_50_ in MDCK cells (36). For evaluating viral thermostability at 37°C, 42°C, and 56°C, the viruses were diluted to 10^8^ TCID_50_/mL or the same HA titers and aliquoted into vials. The viruses were incubated at 37°C and 42°C for 1, 3, and 5 days and for 150 min at 56°C, and the samples were temporarily frozen at −80°C until TCID_50_ titers were determined in MDCK cells (36). The low-pH stability of the viruses was measured by determining viral infectivity after acid treatment. Viruses were incubated in 0.1 M PBS at a pH of 4.6 to 5.5. After incubation at 37°C for 60 min, the viruses were titrated by using TCID_50_ method in MDCK cells (37).

### HI assays and microneutralization assays.

HI assays were performed as described previously (38). Briefly, 25 μL of 2-fold serially diluted antisera was mixed with 25 μL of four HA units of virus and incubated for 15 min at 37°C. Subsequently, 50 μL 0.5% chicken red blood cells was added to each well and incubated for 20 min at 37°C. The HI titer was the highest dilution of the antiserum that completely inhibited hemagglutination. Sera were serially diluted 2-fold and mixed with 200 TCID_50_ of viruses to perform microneutralization assays. After incubation at 37°C for 1 h, the mixture was inoculated into MDCK cells. Neutralization titers were defined as the inverse of the highest serum (39).

### Pathogenicity and contact transmission in chickens.

Groups of five 4-week-old SPF chickens were intranasally inoculated with 10^4^ 50% egg infective dose (EID_50_) of test viruses in 0.2 mL PBS. Four hours postinoculation, five uninfected SPF chickens were placed together with the infected chickens. Mortality was monitored for 2 weeks, cloaca and oropharyngeal swabs of the directly exposed group were collected to determine virus shedding, and all swabs were inoculated with chicken embryos (40). Three additional infected chickens from each group were housed in other isolators and euthanized 3 days postinfection (dpi). The lungs were collected for viral titration in MDCK cells.

### Antigenic analysis and glycan modeling.

The antigenic variation among the viruses was analyzed by cross HI titers, and antigenic cartography was performed according to the methods in Smith et al. (41). HA trimers were generated using the SWISS-MODEL with PDB entries for A/Sichuan/26221/2014 (H5N6) (PDB number: 5hu8). DGalpb1-4DGlcpNAca1-2[DGalpb1-4DGlcpNAcb1-6] DManpb1-3[DGalpb1-4DGlcpNAcb1-2DManpa1-6] DManpb1-4DGlcpNAcb1-4[LFucpa1-6] DGlcpNAcb1-OH was used as a glycan array compound (42, 43). The glycosylated HA trimeric structures were assembled and displayed using PyMol (44).

### Statistical analysis.

Statistical analyses were performed using one-way analysis of variance. GraphPad Prism version 9 software was used to generate the graphs. Statistical significance was set at *P < *0.05.

### Data availability.

The sequence data from this study were deposited in NCBI databases with the accession numbers MZ708707 to MZ708720.
