# Supplementary material for: Emerging of H5N6 Subtype Influenza Virus with 129-Glycosylation Site on Hemagglutinin in Poultry in China Acquires Immune Pressure Adaption
Source: Microbiol Spectr. 2022 Apr 21;10(3):e02537-21. doi: 10.1128/spectrum.02537-21 (PMC9241720; doi:10.1128/spectrum.02537-21)
Supplement: SUPPLEMENTAL FILE 1 — Supplemental material. Download spectrum.02537-21-s001.pdf, PDF file, 0.2 MB [file spectrum.02537-21-s001.pdf]

Supplementary Information

Emerging of H5N6 subtype influenza virus with 129-glycosylation site on hemagglutinin in poultry in China acquires immune pressure adaption

Nuo Xu[1234], Yuwei Wu[1234], Yulian Chen[1234], Yue Li[1234], Yuncong Yin[1234], Sujuan Chen[1234], Huiguang Wu[1], Tao Qin[1234*], Daxin Peng[1234*] and Xiufan Liu[123]

[1] College of Veterinary Medicine, Yangzhou University, Yangzhou, Jiangsu 225009, China

[2]Jiangsu Co-Innovation Center for the Prevention and Control of Important Animal Infectious Disease and Zoonoses, Yangzhou, Jiangsu 225009, China

[3]The International Joint Laboratory for Cooperation in Agriculture and Agricultural Product Safety, Ministry of Education, Yangzhou University, Yangzhou 225009, China

[4]Jiangsu Research Centre of Engineering and Technology for Prevention and Control of Poultry Disease, Yangzhou, Jiangsu 225009, China

*Correspondence: Daxin Peng, Tao Qin; E-mail address: pengdx@yzu.edu.cn, qintao@yzu.edu.cn.

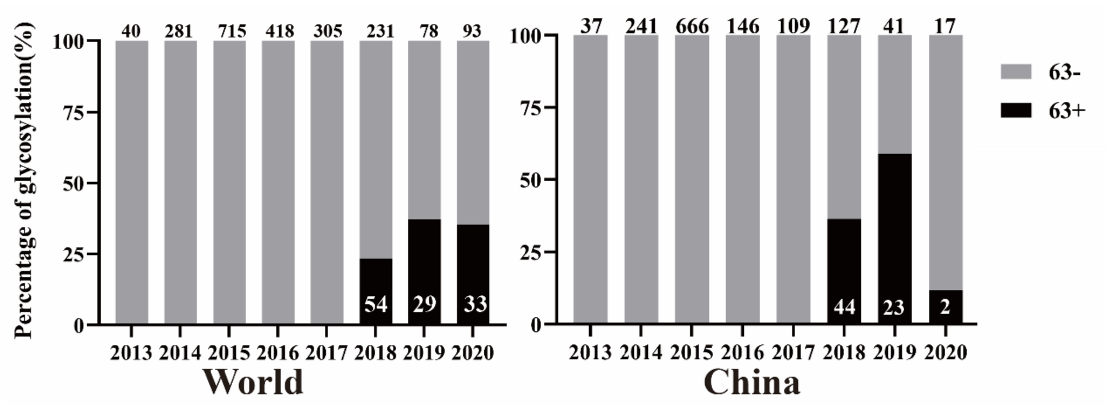

Figure S1 The isolation rates of H5N6 AIVs with the 63-glycosylation site on HA protein in the World and China. The sample size per year were listed at the top of the picture and marked with black, and the number of strains with 63-glycosylation site were marked with white at the bottom of the columns.

Supplementary Table 1 Primers for construction of the recombination HA gene

	Forward primer	Reverse primer
rW HA 129-	TCCAATCATGAAACATCATCA GGAGTGAGCGCA	GATGATGTTTCATGATTGGACCAA GACCTCTTGG
rW HA 63-	TTTTAAAGGATTGTAGTGTAG CTGGATGGCTTCTTG	TACACTACAATCCTTTAAAACCAG AGGTTTCACTCCATT
rB HA 129+	CCCAATCATACATCATCAGGG GTGAGCGCAGCATGCC	CCTGATGATGTATGATTGGGCCAA GAACTCTTGGGGATGAT
rB HA 63+	TTTTAAAGAATTGTAGTGTAG CTGGATGGCTTCTTGGAAA	TACACTACAATTCTTTAAAATCAG AGGTTTCACTCCATT

Supplementary Table 2 Sequence information of phylogenetic tree

Viruses information	GISAID or NCBI ID
A/environment/Shenzhen/25-24/2013(H5N6)	EPI_ISL_161973
/A/chicken/Zhejiang/6C2/2013(H5N6)	EPI_ISL_173670
A/chicken/Shenzhen/1845/2013(H5N6)	EPI_ISL_182061
A/chicken/Shenzhen/1061/2013(H5N6)	EPI_ISL_181666
A/chicken/Hunan/12.07/YYGK114-P/2013(H5N6)	EPI_ISL_198761
A/goose/Eastern/China/S0513/2013(H5N6)	EPI_ISL_208833
A/heron/Guangdong/C1/2013(H5N6)	EPI_ISL_299583
A/duck/Jiangxi/95/2014(H5N6)	EPI_ISL_161972
A/duck/Sichuan/04.27/NBXJ717/2014(H5N6)	EPI_ISL_198866
A/goose/Shandong/11.02/CQ-GS/2014(H5N6)	EPI_ISL_199153
A/goose/Shandong/JNRe-5(H5N6)	EPI_ISL_199410
A/duck/Hunan/01.16/YYFQH128-P/2014(H5N6)	EPI_ISL_200262
A/duck/Sichuan/04.27/NBXJ711/2014(H5N6)	EPI_ISL_200266
A/duck/Guangxi/12.25/GLGX-14-08/2014(H5N6)	EPI_ISL_201370
A/chicken/Fujian/09.16/GT101/2014(H5N6)	EPI_ISL_201376
A/chicken/Fujian/10.08/FZH102/2014(H5N6)	EPI_ISL_201377
A/duck/Jiangxi/09.04/JX-14-11/2014(H5N6)	EPI_ISL_201378
A/chicken/Sichuan/J1/2014(H5N6)	EPI_ISL_202554
A/Duck/Guangdong/SS1A1/2014(H5N6)	EPI_ISL_205959
A/duck/Eastern/China/S0711/2014(H5N6)	EPI_ISL_208836
A/duck/Guangzhou/021/2014	EPI_ISL_224985
A/migratory/waterfowl/Hubei/Chenhu1306/2014	EPI_ISL_234376
A/Copsychus/saularis/Guangdong/SW8/2014	EPI_ISL_234670
A/duck/Hunan/01.16/YYGK0204-P/2014(H5N6)	EPI_ISL_198784
A/goose/Jilin/04.04/SY003-O/2015(H5N6)	EPI_ISL_198757
A/duck/Yunnan/03.16/DQXYL0039-O/2015(H5N6)	EPI_ISL_198882
A/environment/Jiangxi/02.10/NCDZT16/2015(H5N6)	EPI_ISL_198894
A/duck/Guangdong/04.23/DGQTXC202-O/2015	EPI_ISL_199355
A/goose/Yunnan/07.14/DQWCK122/2015(H5N6)	EPI_ISL_200821
A/duck/Fujian/05.07/FZHMDK14-O/2015(H5N6)	EPI_ISL_200822
A/duck/Fujian/05.07/FZHMDK14-O/2015(H5N6)	EPI_ISL_201355
A/Chicken/Guangdong/FG594/2015(H5N6)	EPI_ISL_205964
A/Syrrhaptes/paradoxus/Guangdong/ZH283/2015(H5N6)	EPI_ISL_234679
A/Anser/fabalis/China/P563/2015(H5N6)	EPI_ISL_503031
A/Environment/Guangdong/21109/2015(H5N6)	EPI_ISL_219815
A/Environment/Jiangxi/44908/2015(H5N6)	EPI_ISL_354564
A/Environment/Shenzhen/1/2015(H5N6)	EPI_ISL_205314
A/Environment/Yunnan/DQ53/2015(H5N6)	EPI_ISL_178255
A/Shenzhen/1/2015(H5N6)	EPI_ISL_205313
A/Yunnan/14563/2015(H5N6)	EPI_ISL_178261
A/Guangdong/SZ872/2015(H5N6)	EPI_ISL_206568
A/chicken/Guangdong/10/16/SZLGKQHY/072/2015(H5N6)	EPI_ISL_221885

A/chicken/Hubei/XG18/2015(H5N6)	EPI_ISL_234282
A/chicken/Yunnan/25A/2015(H5N6)	EPI_ISL_195310
A/duck/Hunan/04/14/YYGK0453/1/O/2015(H5N6)	EPI_ISL_200286
A/duck/Zhejiang/02/28/JHZJ/15/04/2015(H5N6)	EPI_ISL_201373
A/environment/Guangdong/04/23 DGQTXC055(H5N6)	EPI_ISL_199395
A/environment/Jiangxi/05/07/NCJD0098C/2015(H5N6)	EPI_ISL_199007
A/duck/Guangdong/01.01/SZSGXJK001-Y/2016(H5N6)	EPI_ISL_221705
A/Chicken/Huizhou/16274/2016(H5N6)	EPI_ISL_278026
A/Goose/Qingyuan/16875/2016(H5N6)	EPI_ISL_278027
A/Quail/Zhanjiang/16887/2016(H5N6)	EPI_ISL_278031
A/Duck/Yunnan/YN-2/2016(H5N6)	EPI_ISL_283702
A/duck/Hunan/7.21/YYGK41Y1-C/2016(H5N6)	EPI_ISL_696707
A/duck/Fujian/11.3/FZHX1132-O/2016(H5N6)	EPI_ISL_707421
A/duck/Fujian/11.3/FZHX1141-C/2016(H5N6)	EPI_ISL_707422
A/duck/Fujian/12.7/FZHX1202-O/2016(H5N6)	EPI_ISL_707425
A/duck/Fujian/12.7/FZHX1218-C/2016(H5N6)	EPI_ISL_707426
A/duck/Guangdong/11.18/SZBJ007-O/2016(H5N6)	EPI_ISL_707455
A/goose/Fujian/10.11/FZHX1002-O/2016(H5N6)	EPI_ISL_707642
A/goose/Fujian/11.3/FZHX1102-C/2016(H5N6)	EPI_ISL_707652
A/goose/Xinjiang/12.18/WLMQXL001-C/2016(H5N6)	EPI_ISL_707662
A/goose/Xinjiang/12.18/WLMQXL002-O/2016(H5N6)	EPI_ISL_707662
A/A/Anhui/33162/2016(H5N6)	EPI_ISL_284650
A/Chicken/Yunnan/YN/11/2016(H5N6)	EPI_ISL_283968
A/Duck/Jiangxi/50446/2016(H5N6)	EPI_ISL_354556
A/Environment/Fujian/52356/2016(H5N6)	EPI_ISL_333636
A/Environment/Jiangxi/50653/2016(H5N6)	EPI_ISL_354550
A/Hubei/29578/2016(H5N6)	EPI_ISL_256213
A/Hubei/29578/2016/x/PR8(H5N6)	EPI_ISL_341293
A/Pavo/cristatus/Jiangxi/JA1/2016(H5N6)	EPI_ISL_257657
A/Shenzhen/TH003/2016/(H5N6)	EPI_ISL_207048
A/chicken/Anhui/MZ34/2016(H5N6)	EPI_ISL_297931
A/chicken/China/CK74/2016(H5N6)	EPI_ISL_499184
A/chicken/Guangdong/GD1602/2016(H5N6)	EPI_ISL_282397
A/chicken/Hubei/ZYSJF11/2016(H5N6)	EPI_ISL_244528
A/chicken/Hunan/12/27/YYGKK148/OC/(H5N6)	EPI_ISL_699454
A/duck/Jiangxi/11/29/NCDZT71H2/O/(H5N6)	EPI_ISL_707546
A/enviroment/Guangdong/F4/2016(H5N6)	EPI_ISL_266819
A/goose/Guangdong/QY01/2016(H5N6)	EPI_ISL_272740
A/poultry/China/XY918/4/2016(H5N6)	EPI_ISL_282408
A/Ostrich/Guangxi/GX-1/2017/(H5N6)	EPI_ISL_283970
A/Quail/Guangxi/GX-2/2017/(H5N6)	EPI_ISL_283971
A/Duck/Guangdong/PO17281256/MZH/2017-8-21(H5N6)	EPI_ISL_340789
A/Env/Guangdong/C172790591/ZHQ/2017-5-8(H5N6)	EPI_ISL_340848
A/Env/Guangdong/C17272335/SHG/2017-5-16(H5N6)	EPI_ISL_340853

A/Env/Guangdong/EN17284259/YJ/2017-7-12(H5N6)	EPI_ISL_340856
A/Env/Guangdong/C17280709/HZH/2017-5-2(H5N6)	EPI_ISL_340858
A/Greylag/goose/Hunan/1/2017(H5N6)	EPI_ISL_400492
A/duck/China/0936/2017(H5N6)	EPI_ISL_503024
A/duck/Jiangxi/11.29/NCNP22D3-OC/2017(H5N6)	EPI_ISL_696686
A/duck/Jiangxi/11.29/NCNP39D3-OC/2017(H5N6)	EPI_ISL_696703
A/duck/Hunan/11.30/YYGK75E3-OC/2017(H5N6)	EPI_ISL_696740
A/duck/Fujian/10.26/FZHX0034-C/2017(H5N6)	EPI_ISL_697188
A/duck/Fujian/1.17/FZHX0123-C/2017(H5N6)	EPI_ISL_707384
A/duck/Fujian/10.26/FZHX0002-O/2017(H5N6)	EPI_ISL_707414
A/duck/Hunan/03.24/YYGK174M2-C/2017(H5N6)	EPI_ISL_707478
A/duck/Hunan/1.17/YYGKK80-OC/2017(H5N6)	EPI_ISL_707493
A/duck/Hunan/11.30/YYGK59E3-OC/2017(H5N6)	EPI_ISL_707500
A/duck/Hunan/11.30/YYGK62E3-OC/2017(H5N6)	EPI_ISL_707501
A/duck/Jiangxi/01.11/NCNP101G2-OC/2017(H5N6)	EPI_ISL_707533
A/duck/Guangdong/G1378/2018(H5N6)	EPI_ISL_314857
A/Jiangsu/1/2018(H5N6)	EPI_ISL_332402
A/Pavo/cristatus/China/JS01/2018(H5N6)	EPI_ISL_370415
A/mink/Eastern/China/006/2018(H5N6)	EPI_ISL_502201
A/mink/Eastern/China/571/2018(H5N6)	EPI_ISL_502208
A/duck/Guizhou/7.27/ZYLJJ017-O/2018(H5N6)	EPI_ISL_696807
A/duck/Guizhou/8.26/ZYLJJ016-O/2018(H5N6)	EPI_ISL_696813
A/chicken/Guizhou/10.28/ZYLJJ008-C/2018(H5N6)	EPI_ISL_696836
A/pigeon/Hainan/1.14/HKPL006-C/2018(H5N6)	EPI_ISL_696843
A/goose/Fujian/3.15/FZHX0001-O/2018(H5N6)	EPI_ISL_696991
A/goose/Fujian/3.15/FZHX0007-O/2018(H5N6)	EPI_ISL_696992
A/goose/Fujian/3.15/FZHX0005-O/2018(H5N6)	EPI_ISL_697214
A/chicken/Hunan/02.06/YYGK37J3-OC/2018(H5N6)	EPI_ISL_699429
A/duck/Guizhou/8.26/ZYLJJ017-O/2018(H5N6)	EPI_ISL_707465
A/duck/Hunan/01.12/YYGK82H3-OC/2018(H5N6)	EPI_ISL_707475
A/duck/Hunan/1.12/YYGK68H3-OC/2018(H5N6)	EPI_ISL_707489
A/duck/Hunan/2.06/YYGK78J3-OC/2018(H5N6)	EPI_ISL_707524
A/duck/Jiangxi/2.28NCNP23K3-OC/2018(H5N6)	EPI_ISL_707554
A/goose/Fujian/3.15/FZHX0008-C/2018(H5N6)	EPI_ISL_707653
A/Chicken/Suzhou/j5/2019(H5N6)	EPI_ISL_353285
A/Chicken/Suzhou/j6/2019(H5N6)	EPI_ISL_353618
A/chicken/Shandong/01/26/TAWL003/O/2019(H5N6)	EPI_ISL_697901
A/chicken/Shandong/01/26/TAWL011/O/2019(H5N6)	EPI_ISL_697903
A/chicken/Shandong/01/26/TAWL017/O/2019(H5N6)	EPI_ISL_697904
A/JiangsuNanjing/1128/2020(H5N6)	EPI_ISL_718266
A/Muscovy/duck/China/FJFZ21/2020(H5N6)	EPI_ISL_833248
A/Mute/swan/Xinjiang/4/2020(H5N6)	EPI_ISL_418172
Whooper/Whooper/swan/Xinjiang/10/2020/H5N6	EPI_ISL_418178
Whooper/Whooper/swan/Xinjiang/1/2020/H5N6	EPI_ISL_418169

A/Whooper/swan/Xinjiang/2/2020(H5N6)	EPI_ISL_418170
A/Whooper/swan/Xinjiang/3/2020(H5N6)	EPI_ISL_418171
A/Whooper/swan/Xinjiang/7/2020(H5N6)	EPI_ISL_418175
A/Whooper/swan/Xinjiang/8/2020(H5N6)	EPI_ISL_418176
A/Whooper/swan/Xinjiang/9/2020(H5N6)	EPI_ISL_418177
A/duck/Hyogo/1/2016(H5N6)/clade2.3.4.4e	EPI_ISL_293500
A/duck/Vietnam/LBM360c1-4-1/2013/H5N6	EPI_ISL_173146
A/Fujian-Sanyuan/21099/2017/(H5N6)clade2.3.4.4b	EPI_ISL_304404
A/Guangdong/18SF020/2018/(H5N6)clade2.3.4.4h	EPI_ISL_337274
A/Guizhou/1/2013/(H5N1)clade2.3.4.2	EPI_ISL_135216
A/gyrfalcon/Washington/41088-6/2014(H5N8)clade2.3.4.4c	EPI_ISL_173878
A/Hubei/29578/2016b(H5N6)/clade2.3.4.4d	EPI_ISL_256213
A/Sichuan/26221/2014(H5N6)/clade2.3.4.4a	EPI_ISL_163493
Re-11.	No ID
A/chicken/Bangladesh/2011(H5N1)clade2.3.4.2	EPI_ISL_240029
A/chicken/Vietnam/NCVD-15A59/2015(H5N6)clade2.3.4.4f	EPI_ISL_244518
A/barn/swallow/Hong/Kong/D10-1161/2010(H5N1)clade2.3.2.1b	EPI_ISL_137877
A/chicken/Ghana/20/2015(H5N1)clade2.3.2.1f	EPI_ISL_223929
A/chicken/Guizhou/1153/2016/clade/(H5N1)clade2.3.2.1d	EPI_ISL_503628
A/common/magpie/Hong/Kong/5052/2007(H5N1)clade2.3.2.1	EPI_ISL_25690
A/duck/Bangladesh/19097/2013(H5N1)clade2.3.2.1a	EPI_ISL_151729
A/goose/Jiangsu/TX0202/2018(H5N6)	MZ708707
A/goose/Wuhu/WH0109/2019/(H5N6)	MZ708708
A/goose/Jiangsu/YZG161111/2016/(H5N6)	MZ708709
A/chicken/Jiangsu/HJ1107/2017(H5N6)	MZ708711
A/chicken/Jiangsu/LA1013/2017(H5N6)	MZ708712
Re-8	EPI_ISL_202553
A/goose/BaoYing/BY160916/2016(H5N6)	MZ708710
A/Green pheasant/Hunan/10/2015 clade2.3.4.4g	EPI_ISL_400485
A/Common pheasant/Hunan/11/2015 clade2.3.4.4g	EPI_ISL_400486
